# Supplementary material for: Primosomal protein PriC rescues replication initiation stress by bypassing the DnaA-DnaB interaction step for DnaB helicase loading at oriC
Source: eLife. 2025 May 29;13:RP103340. doi: 10.7554/eLife.103340 (PMC12122000; doi:10.7554/eLife.103340)
Supplement: Figure 3—figure supplement 1—source data 1. — Colored protein size markers were used. Each lane is labeled as in the main text. [file elife-103340-fig3-figsupp1-data1.zip › Figure 3-figure supplement 1A-source data 1.pdf]

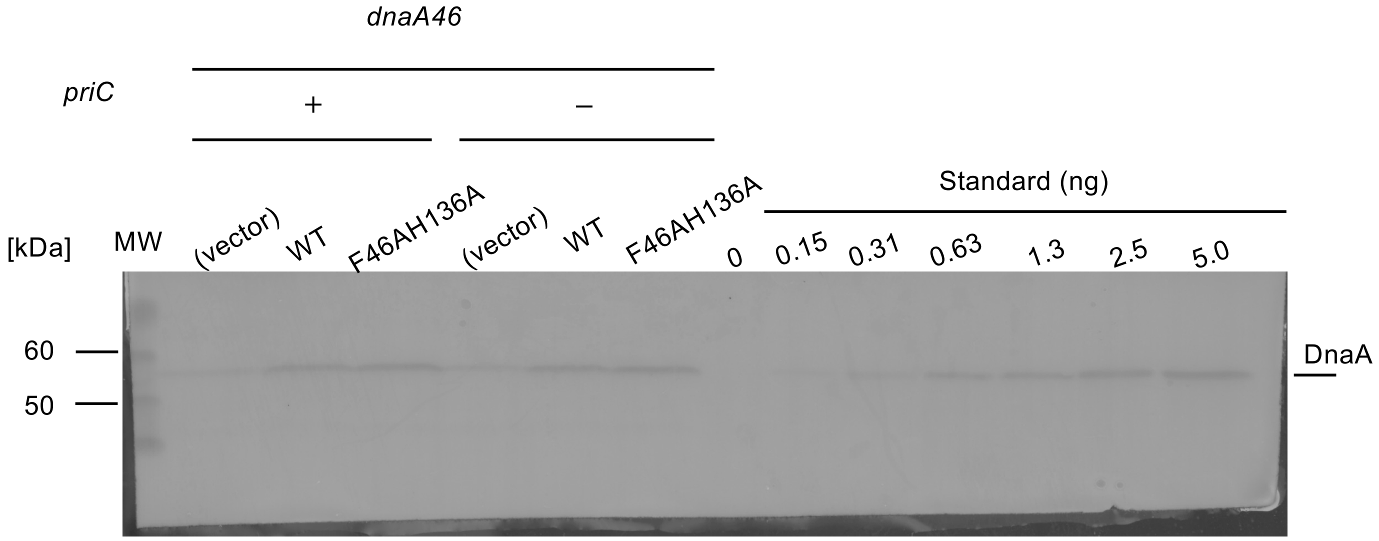
dnaA46
priC
+
–
Standard (ng)
[kDa]
MW
(vector)
WT
F46AH136A
(vector)
WT
F46AH136A
0
0.15
0.31
0.63
1.3
2.5
5.0
60
50
DnaA
