# Supplementary figures and images for: Primosomal protein PriC rescues replication initiation stress by bypassing the DnaA-DnaB interaction step for DnaB helicase loading at oriC
Source: eLife. 2025 May 29;13:RP103340. doi: 10.7554/eLife.103340 (PMC12122000; doi:10.7554/eLife.103340)

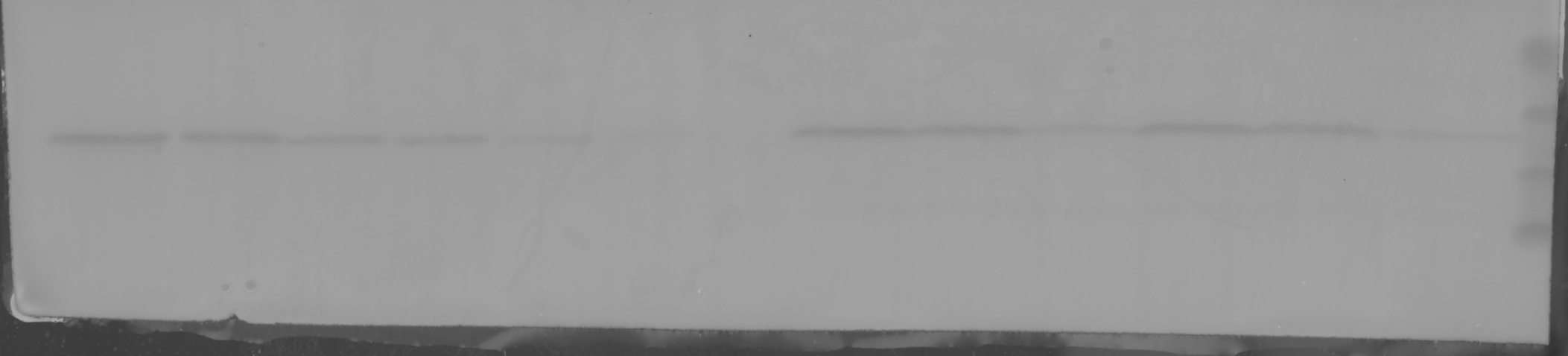

Supplement: Figure 3—figure supplement 1—source data 2. [file elife-103340-fig3-figsupp1-data2.zip › Figure 3-figure supplement 1A-source data 2.tiff]

Panel A

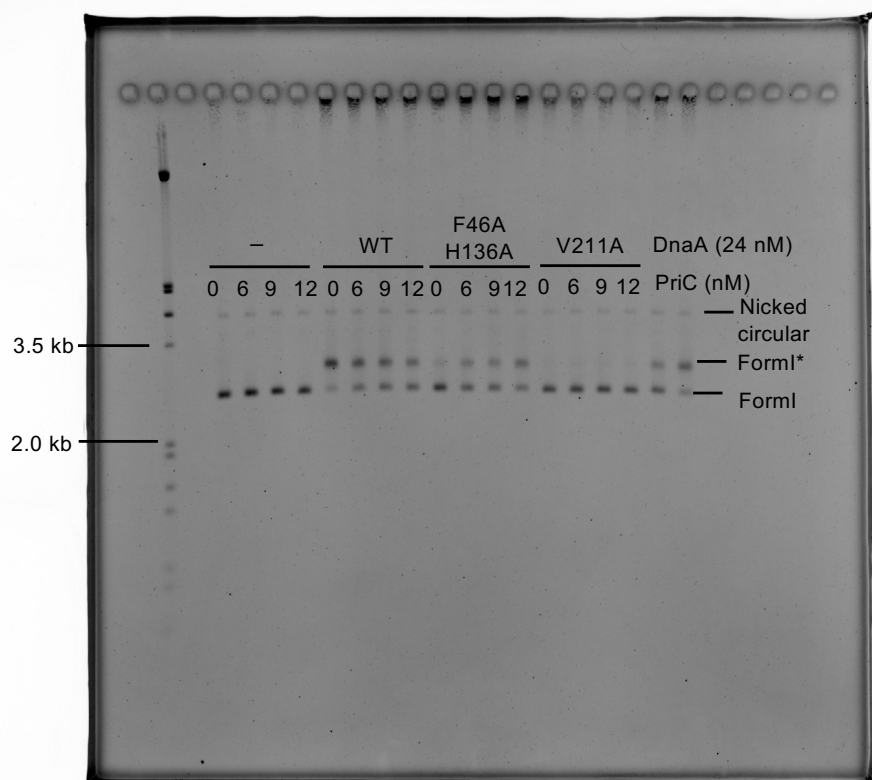

Panel C

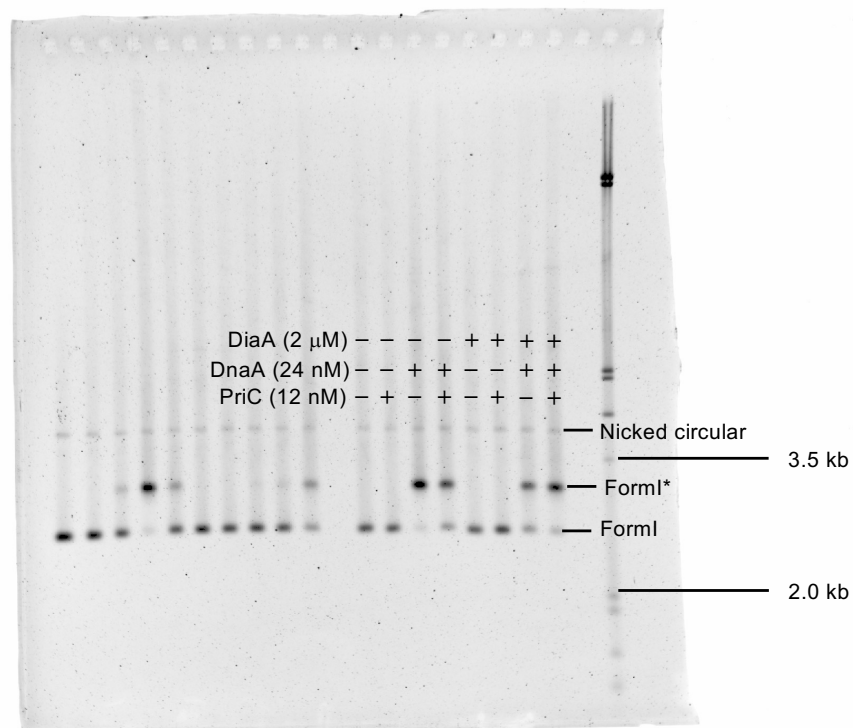

Supplement: Figure 7—source data 1. — Lambda DNA markers were employed. Each lane shown is labeled as that in the main text. [file elife-103340-fig7-data1.zip › Figure 7AC-source data 1.pdf]

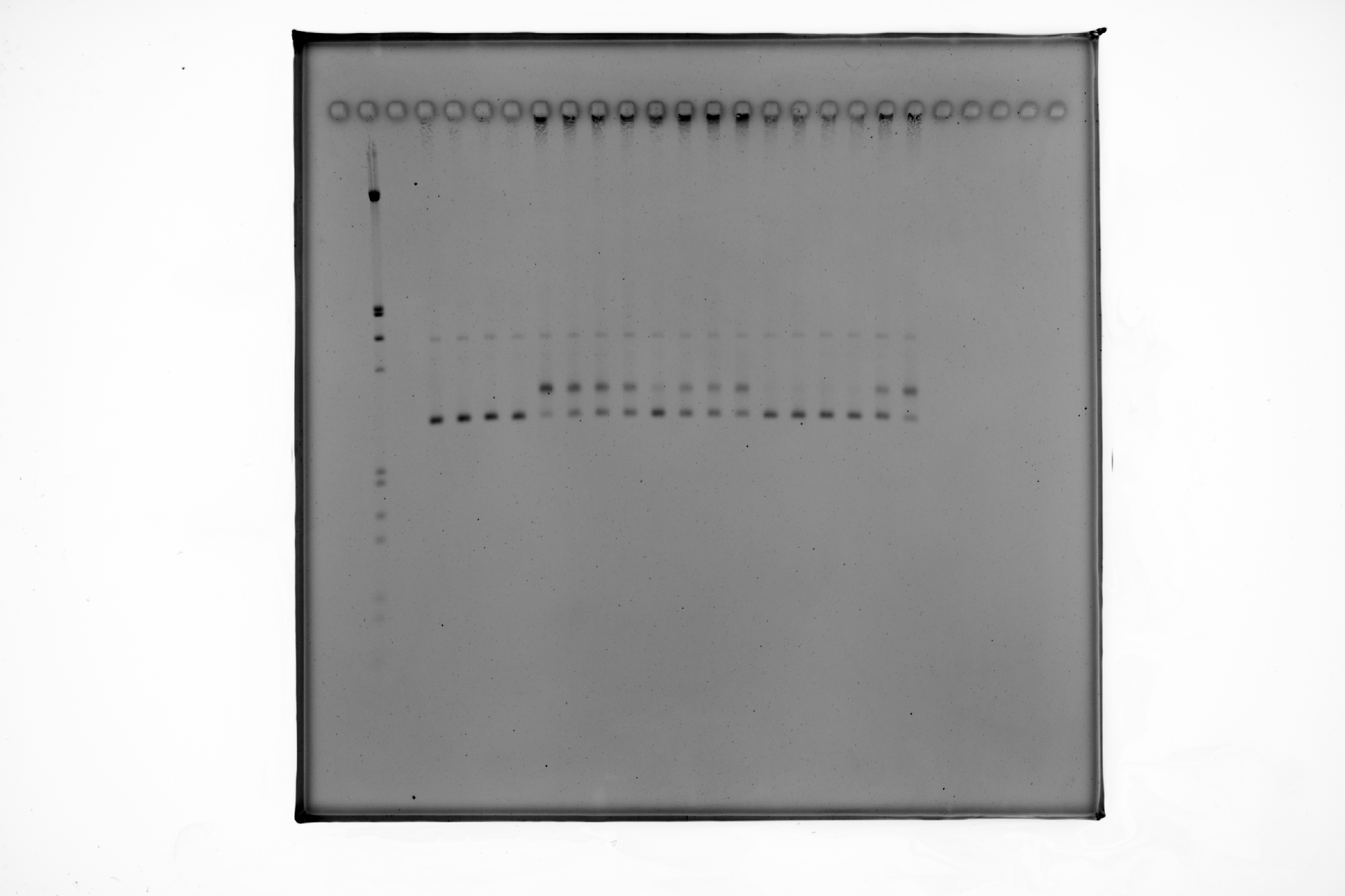

Supplement: Figure 7—source data 2. [file elife-103340-fig7-data2.zip › Figure 7A-sourse data 2.tif]

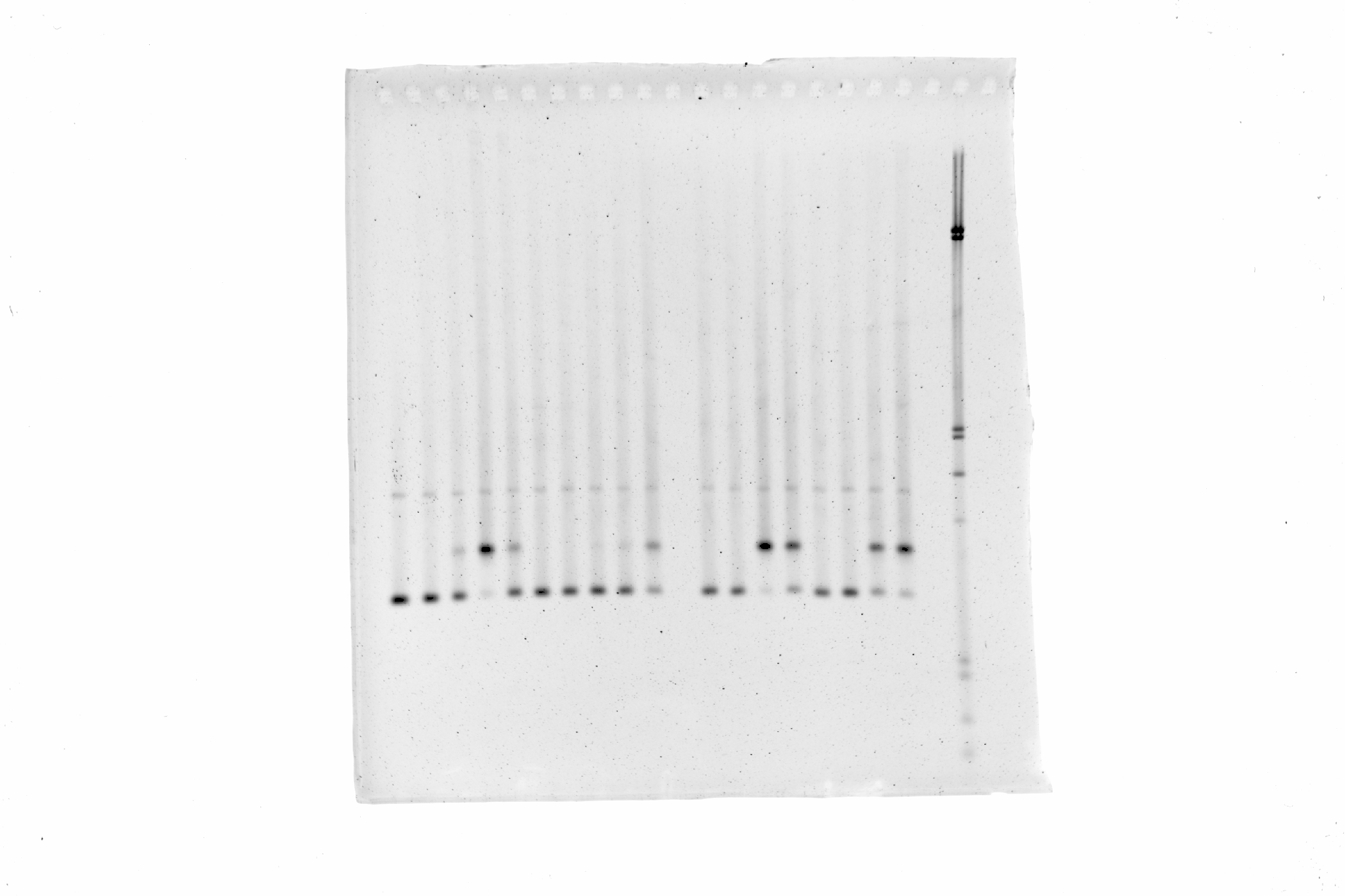

Supplement: Figure 7—source data 3. [file elife-103340-fig7-data3.zip › Figure 7C-sourse data 2.tif]

Panel A

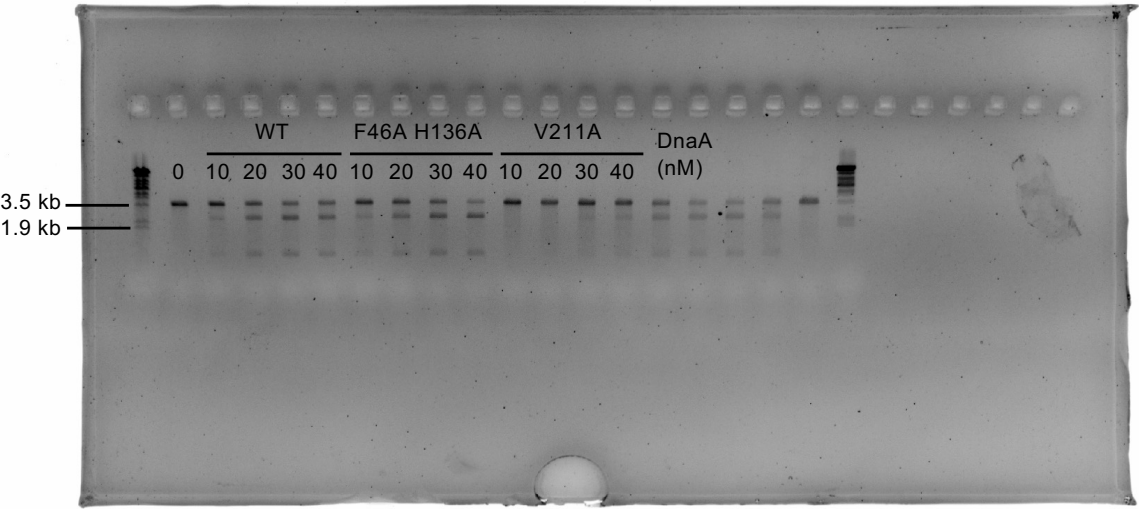

Panel C

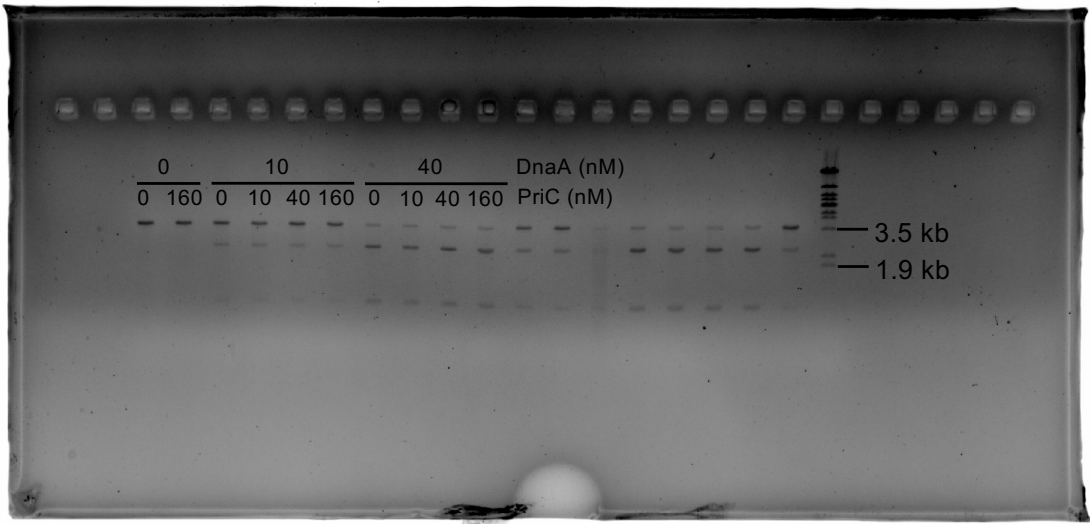

Supplement: Figure 7—figure supplement 1—source data 1. — Lambda DNA markers were employed. Each lane is labeled as in the main text. [file elife-103340-fig7-figsupp1-data1.zip › Figure 7-Figure Supplement 1AC-Source data 1.pdf]

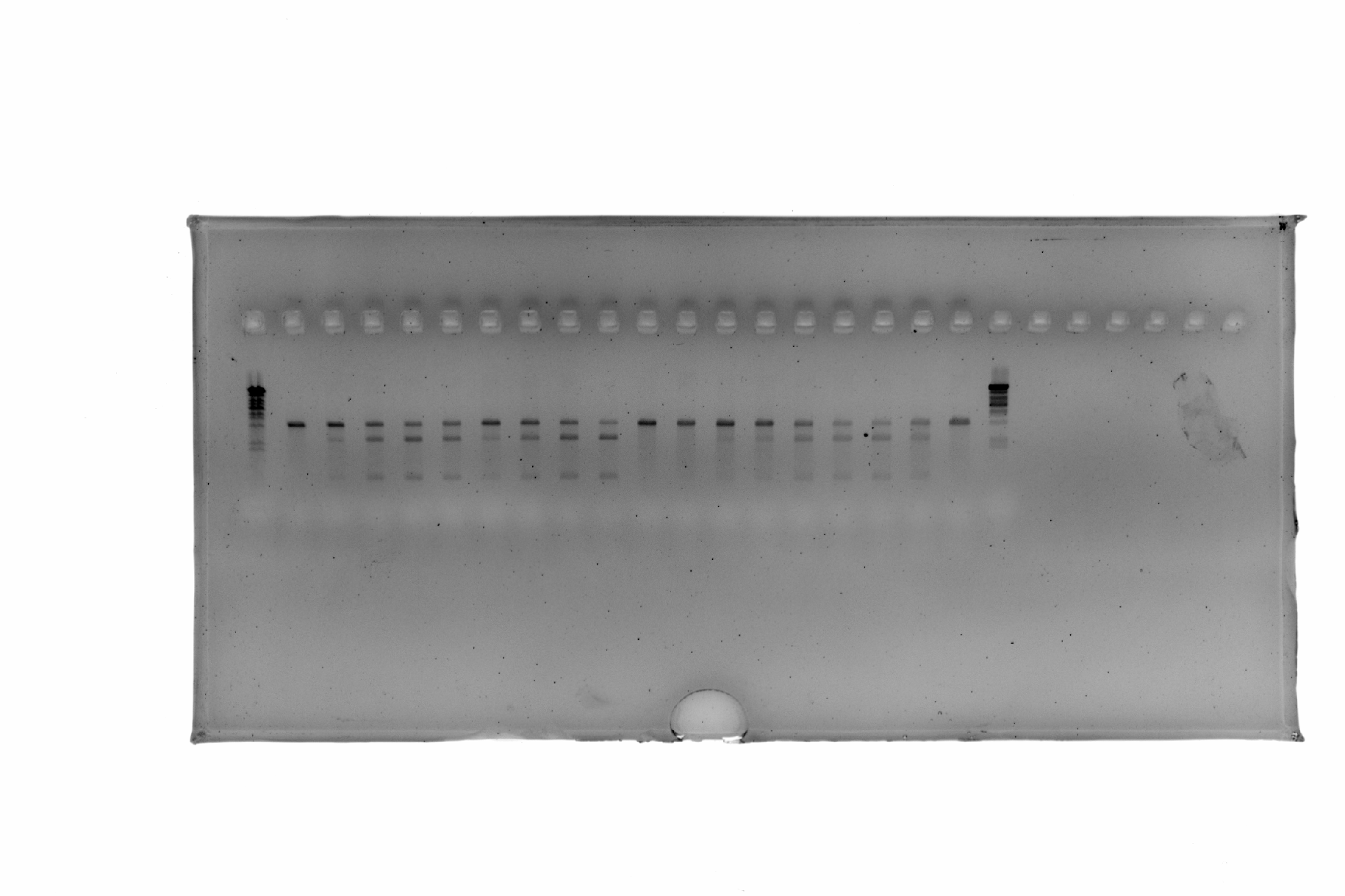

Supplement: Figure 7—figure supplement 1—source data 2. [file elife-103340-fig7-figsupp1-data2.zip › Figure 7-Figure Supplement1A-Source data 2.tif]

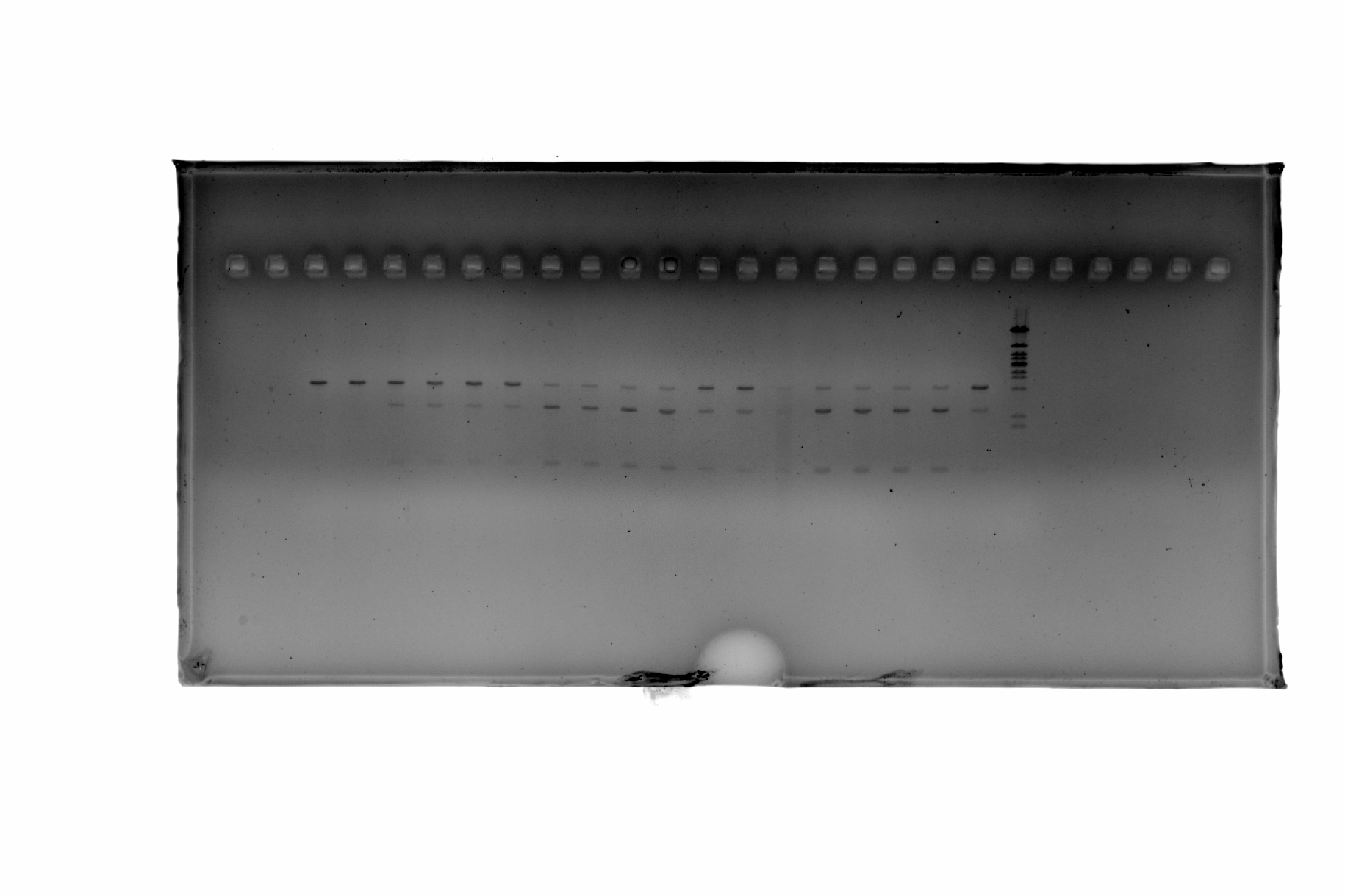

Supplement: Figure 7—figure supplement 1—source data 3. [file elife-103340-fig7-figsupp1-data3.zip › Figure 7-Figure Supplement1C-Source data 2.tif]
